# Survey of Hatching Spines of Bee Larvae Including Those of *Apis mellifera* (Hymenoptera: Apoidea)

**DOI:** 10.1093/jisesa/iex060

**Published:** 2017-07-24

**Authors:** Jerome G. Rozen, Corey Shepard Smith, James H. Cane

**Affiliations:** 1 Division of Invertebrate Zoology, American Museum of Natural History, Central Park West at 79th St., New York, NY 10024 (rozen@amnh.org, csmith@amnh.org); 3 USDA–ARS Bee Biology and Sytematics Laboratory, Pollinating Insect Research Unit, Utah State University, Logan, UT 84322-5310 (jim.cane2@gmail.com)

**Keywords:** hatching spine, eclosion, bee, *Apis mellifera*

## Abstract

This article explores the occurrence of hatching spines among bee taxa and how these structures enable a larva on hatching to extricate itself from the egg chorion. These spines, arranged in a linear sequence along the sides of the first instar just dorsal to the spiracles, have been observed and recorded in certain groups of solitary and cleptoparasitic bee taxa. After eclosion, the first instar remains loosely covered by the egg chorion. The fact that this form of eclosion has been detected in five families (Table 1 identifies four of the families. The fifth family is the Andrenidae for which the presence of hatching spines in the Oxaeinae will soon be announced.) of bees invites speculation as to whether it is a fundamental characteristic of bees, or at least of solitary and some cleptoparasitic bees. The wide occurrence of these spines has prompted the authors to explore and discover their presence in the highly eusocial *Apis mellifera* L. Hatching spines were indeed discovered on first instar *A. mellifera.* The honey bee hatching process appears to differ in that the spines are displayed somewhat differently though still along the sides of the body, and the chorion, instead of splitting along the sides of the elongate egg, seems to quickly disintegrate from the emerging first instar in association with the nearly simultaneous removal of the serosa that covers and separates the first instar from the chorion. Unexpected observations of spherical bodies of various sizes perhaps containing dissolving enzymes being discharged from spiracular openings during hatching may shed future light on the process of how *A. mellifera* effects chorion removal during eclosion. Whereas hatching spines occur among many groups of bees, they appear to be entirely absent in the Nomadinae and parasitic Apinae, an indication of a different eclosion process.

The term “hatching spine” was used by Wigglesworth (1947) for a variety of cuticular structures sometimes found on the embryonic cuticle or other times on the cuticle of the first instars of insects, which are used to cut through the embryonic cuticle or chorion at the time of hatching from the egg. Sometimes termed “egg bursters,” they have been observed in many groups of insects but, until recently, have gone unnoticed by melittologists. The purpose of this article is to point out that hatching spines in the form of very small spicules are widely dispersed among bee taxa and, furthermore, appear to be a signature marker of the identity of a first larval instar for many (but not all) taxa, often necessary for distinguishing first instars from later stages.

This study is presented in three parts: (1) a survey of hatching spines and hatching processes in solitary and cleptoparasitic bees, (2) an investigation of hatching spines in first larval instars of the highly eusocial bee, *Apis mellifera*, and (3) an investigation into the egg hatching process of species of Apidae the first instar of which do not exhibit hatching spines.

## Methods

All SEM micrographs were captured using a Hitachi S5700 in the Microscopy and Imaging Facility of the American Museum of Natural History. All figures (except for Figs. 4–6) are SEM micrographs of *A. mellifera*, oriented with anterior ends toward the left. Larval specimens had been preserved and stored in Kahle’s Solution [acetic acid (glacial) 10%; formalin 10%; water 25%, ethyl alcohol (74%) 55%].

### 

#### Hatching Spines of Solitary and Cleptoparasitic Bees

Table 1 is a taxonomically arranged survey of the literature dealing with larval eclosion among solitary and cleptoparasitic bees. Among first instars of solitary and some cleptoparasitic bees (i.e., nonsocial bees), hatching spines appear as a row of sharp-pointed, minute spicules that extend along both sides of the body just above the spiracles of the first instar. In most cases they are a narrow, continuous, linear row of spicules on the surface of each segment above but close to the spiracular line. Because of their small size, they are rarely identifiable when viewed by stereomicroscope, probably accounting for their being overlooked by numerous researchers studying late embryogenesis and eclosion. Further, they may be obscure because the widespread practice of immersing hatching eggs in paraffin oil for microscopic observation tends to render the spines transparent (DuPraw 1963).

**Table 1.** Annotated systematic accounts of larval eclosion in solitary and cleptoparasitic bees (Hymenoptera: Apoidea) cleptoparasitic taxa identified by gray shading; non-shaded taxa solitary

Taxon	Reference and annotation
**Stenotritidae**	
**Colletidae**	
**Colletinae**	
*Colletes kincaidii* Cockerell	Torchio et al. (1988). Fluid ingestion; tracheal system fills with air; then embryo rotation; chorion splits along both sides; second instar starts to feed
Hylaeinae *Hylaeus leptoceph-*	Torchio (1984). “4th instar finishes feeding; 5th defecates.” *alus* (Morawitz)
**Halictidae**	
Nomiinae	
*Nomia melanderi* Cresson	Hackwell and Stephen (1966). Embryo rotation; first instar chorion discovered; head widths of 1st and 2nd instars close in size
**Melittidae**	
**Megachilida**e	
Osmiini	
*Osmia lignaria*	Torchio (1989a). After rotation, chorion splits along spiracular line; then splits along dorsal mid line and 2nd instar appears
*propinqua* Cresson	
*O. californica*	Torchio (1989a). Same as above.
Cresson	
*O. m. montana*	Torchio (1989a). Presumably same as above
Cresson	
Anthidiini	
*Stelis montana* Cresson	Torchio (1989b). Rotation; tracheae gas fill; chorion splits laterally toward rear with appearance of molting fluid on dorsum; chorion and first instar exuviae removed simultaneously before 2nd instar feeds on provisions
Dioxiini	
*Dioxys cinctus* (Jurine)	Rozen and Özbek (2004). First instar pharate, exuviae found with cast chorion
Megachilini	
*Megachile rotundata* (Fabricius)	Trostle and Torchio (1994). Rotation, swelling, splitting, chorion covered [hatching = removal of chorion]
*M. apicalis* Spinola	Similar to above
*M. pugnata* Say	Frolich and Parker (1983). Lateral split of chorion at level of spiracle; chorion seemed to dissolve, then started to feed
*Coelioxys chichi-mecca* Cresson	Rozen et al. (2010). 1st instar in chorion, only 2nd instar out, darkly pigmented
**Apidae**	
Nomadinae	
Epeolini:	
*E. compactus*	Torchio and Burdick (1988). Rotation 180°, emerges through front of egg
Cresson	
*T. dacotensis* (Stevens)	Torchio (1986). Rotation 180°; no splitting of chorion along sides; exit through front of egg
Biastini:	
*Biastes emarginatus* (Schenck)	Rozen et al. (2009). Ventral and lateral cleat-like spicules
Apinae	
Eucerini	
*Svastra o. oblique* (Say)	Rozen (1964). Splitting of chorion along spiracular line identified for first time
Tapinotaspidini	
*M. haemorrhoidalis* (Smith)	Rozen et al. (2006). Complete understanding; “granules” identified as spicules which probably serve as splitting mechanism
Tetrapediini	
*T. diversipes* Klug	Alves-dos-Santos, et al. (2002). Integument of first instar “with linear row of granules” on each side of body
Anthophorini	
*Anthophora u. urbana* Cresson	Torchio and Trostle (1986). Rotation, fluid ingestion, dissolution of chorion around each spiracle; then splits along dorsal midline; first instar then feeds; instars not counted
*A. occidentalis*	Torchio (1986). Like *A. u. urbana*
Cresson	
*A. flexipes* Cresson	Torchio and Youssef (1968). 1st stage larva “fed upon its provision immediately after hatching.”
*A. peritomae*	Torchio (1971). Splitting of chorion along pleural region above spicules
Cockerell	
*A. braunsiana*	Rozen (1969). Shiny strip along each side
Centridini	
*C. flavofasciata* Friese	Rozen et al. (2011). SEM micrographs (Figs. 15, 17, and 18). document hatching spicules splitting chorion of hatching first instar, and photomicrograph (Fig. 20) showing removal of chorion attached to first instar exuviae with the molt to second instar
*Centris bicornuta* Mocsáry	Rozen (2017). Eclosion of this species as described for *C. flavofasciata* above; hatching spines well illustrated (Fig. 5)
*Epicharis picta* (Smith) & *E. nigrita* (Friese)	Gaglianone et al. (2015: 406). Same mode of development and behavior and with both the analysis of the chorion and cuticle of “the first larval stage (evident due to the presence of spiracles and spicules) was attached to the chorion of the egg, indicating that the hatched larva represented the second larval stage.”
*Epicharis albofasciata S*mith	Rozen (2016; 2017). Larval eclosion interpreted to be like that of *M. haemorrhoidalis* and that of *C. flavofasciata* and *C. bicornuta*
Melectini	
*Xeromelecta californica* (Cresson)	Torchio and Trostle (1986). Rotation, ingestion of embryonic fluid; dorsal chorion around head splits
*Zacosmia maculate* (Cresson)	Torchio and Youssef (1968). 1st stage larva tears open anterior tip of egg probably with aid of head spines
*Euglossini*	
*Exaerete smaragdina* (Guérin-Ménvill)	Garófalo and Rozen (2001). “Granules” present on first instar exuviae

The presence of hatching spines in bees was initially detected in *Tetrapedia diversipes* Klug (Apidae: Tetrapediini) by Alves-dos-Santos *et al.* (2002: p. 28) as “a linear row of granules” along the two sides of the body of a hatching larva. The authors concluded that the first larval instar was pharate within the chorion before the chorion ruptured above the spiracular line. No function was ascribed to the “granules”. Instead, it was hypothesized that the rupturing resulted solely from increase in body size caused by ingestion of amniotic fluid. The chorion was shed with the first instar exuviae, allowing the recognition of the second instar, which was actively feeding.

Four years later, most of the same authors used SEM to examine egg hatching of *Monoeca haemorrhoidalis* (Smith) and related taxa (Apidae: Tapinotaspidini) (Rozen et al. 2006: Figs. 14 and 15). They determined that the “granules” above the spiracular line were in fact extremely small, sharp-pointed spicules. Furthermore, when food provisions were colored with dye, the pharate first instar was seen to ingest not only amniotic fluid but also liquid from the surface of the provisions possibly through the micropylar opening on the chorion. Because of the spicules’ stout bases, sharply pointed apices, and their position just above the spiracular line, the authors at first thought that the spicules may be a tearing mechanism causing the chorion to split along this line not only in *M. haemorrhoidalis* but in many groups of solitary and cleptoparasitic bees (see Table 1). This explanation accords with a good many observations of lateral chorion splitting among a range of taxa by various authors. Good illustrations of the hatching process in a solitary bee can be found in Rozen et al. (2011: Figs. 15–20) for *Centris flavofasciata* Friese. See Table 1 for references to solitary and cleptoparasitic bees exhibiting features of this eclosion process. While we first thought that hatching spines alone were responsible for a mechanical splitting along the two sides of the chorion of solitary and some cleptoparasitic bee eggs, we now think that a hatching enzyme may also play a role in this process, as has been suggested by others (e.g., DuPraw 1967, Torchio 1984).

The information in Table 1 suggests that, among solitary and some cleptoparasitic bees, the procedure of hatching when fully investigated will be as follows: First, ingestion of amniotic fluid and fluid from provisions causes body swelling, which in turn results in the splitting of the chorion above the spiracular line on both sides of the first instar’s body presumably with the aid of a hatching enzyme. The first instar’s existence is brief; it is almost, if not always, loosely covered to some extent by the egg chorion thereafter. It ingests little or no pollen from the provisions. This then is our current understanding; further studies will be required to confirm or modify this assessment.

#### Hatching Spines of *A. mellifera*

Because accounts of larval eclosion of *A. mellifera* L. (Apidae: Apini) have not mentioned hatching spines (e.g., Nelson 1915, DuPraw 1967), we undertook an SEM examination of several hatching worker eggs of this species and quickly detected spicules clustering mostly just above the spiracular line on both sides of the body (Figs. 3, 4, 6–8, 12, 14, 15). When highly magnified, these spines (Figs. 14 and 15) closely match the structure and appearance of those observed among nonsocial taxa (e.g., Rozen 2017: Fig. 5) but their arrangement on the body surface is distinctive in that they appear to form a loose band mostly just above the spiracles (Figs. 6–8) rather than a linear series (Rozen et al. 2006: Figs. 14 and 15) spanning the body segments. Because of their position above and close to the line of spiracles, their individual appearance, and their function in ridding the body of chorion (discussed below), they are likely homologous with the hatching spines of nonsocial bees identified above.

However, there is no evidence that the egg chorion of a honey bee splits along the sides, as found among nonsocial bees; rather, it appears (through a series of micrographs of various specimens: Figs. 5, 16, 19–21, 26, and 28) to disassemble from the body surface presumably with the assistance of the sharp spicules (i.e., hatching spines), leaving behind a thin, transparent membrane (presumably the serosa amnion), which also then disintegrates.

**Fig. 1.**
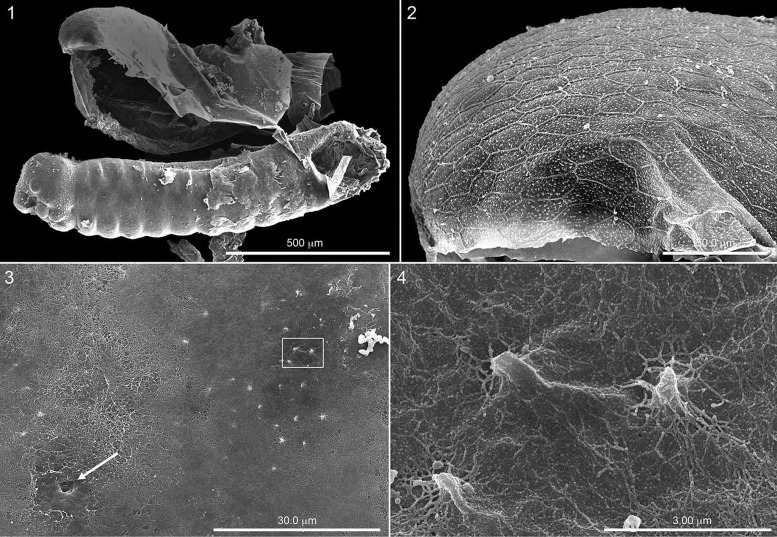
*Apis mellifera* egg that had accidently been broken while being mounted on SEM stub, so that chorion above) had been torn off exposing, in lateral view, anterior end to left, the embryo covered by a thin, transparent, cuticular-like serosa amnion (not visible) covered by a fibrous layer revealing the head shape and segmentation of the first instar. Fig. 2. Close-up of anterior end of the chorion clearly revealing reticulated surface pattern. Fig. 3. Surface of fibrous layer in the vicinity of spiracle (arrow) on third thoracic segment in association with apices of hatching spines. Fig. 4. Close-up of rectangle in Fig. 3, showing fibers clinging to spines as well as to invisible serosa.

To understand the anatomy of a honey bee egg, we examined an egg (Fig. 1) from which much of the chorion had been accidently removed from the embryo when mounted on an SEM stub. This permitted a view of a free chorion clearly identifiable because of its anterior reticulate patterning (Fig. 2). The chorion was separated from the developing first instar by the membranous serosa with a minutely fibrous outer surface (Figs. 3, 4, and 28). The fibrous covering of the outer surface was absent around the two thoracic spiracles on the one side as well as around a number of abdominal spiracles, thereby exposing a smooth, presumably transparent surface and the pit-like indentations to the spiracles (arrow, Fig. 3). Elsewhere, but especially above the spiracular line, the sharp apices of the hatching spines protruded through the fibers (Figs. 3 and 4). As demonstrated in Figs. 14, 15, and 29, all spicules are covered by the serosa, which envelopes and closely adheres to the entire developing first instar, including the spiracular openings at the bottom of the spiracular pits (Figs. 12 and 13). The small, presumably unclogged holes visible in the SEM image of the integument at the bottom of the pits (Figs. 13 and 18) is presumably not be covered by this transparent serosa.

**Fig. 5.**
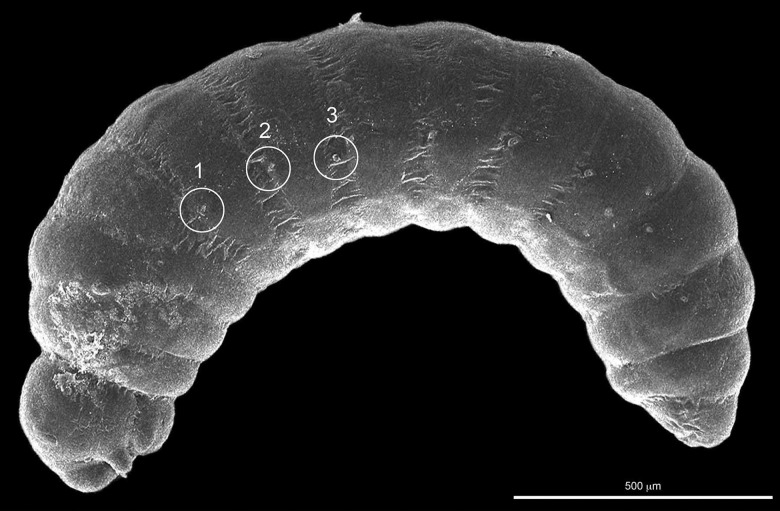
First instar still covered by serosa showing position of spiracles and small quantity of chorion on thorax and head, lateral view. Position of abdominal spiracles 1, 2, and 3 circled on micrograph. Distribution of hatching spines not visible because obscured by serosa.

**Figs. 6–8.**
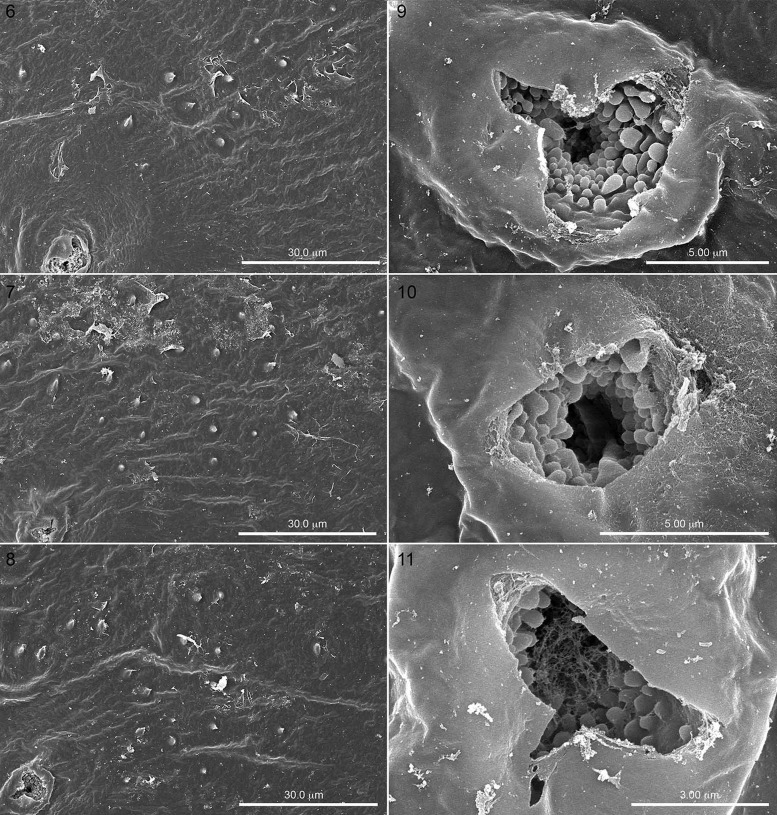
Close-ups of spiracles (lower left corners) and hatching spines of abdominal segments 1, 2, and 3, demonstrating that a band of white-tipped spines extends along the length of the larva’s thorax and abdomen. Figs. 9–11. Partly opened spiracles of abdominal segments 4, 6, and 8 showing small spheroids developing in atria.

**Fig. 12.**
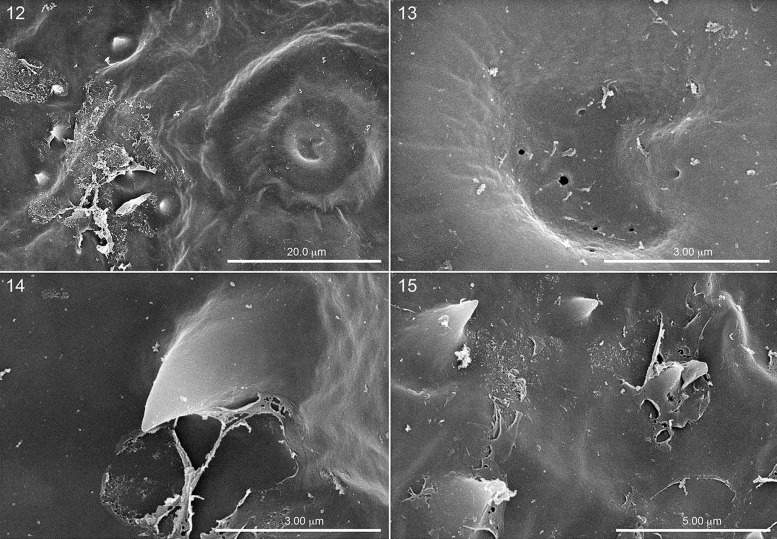
Spiracular area on another first instar still covered by serosa so that spiracular aperture covered. Fig. 13. Close-up of closed aperture showing small circular openings in serosa that perhaps allows discharge of hatching enzyme. Fig. 14. Close-up of single hatching spine showing shreds of serosa, strongly suggesting that spines are responsible for shredding of serosa and involved with dissolution of chorion. Fig. 15. Close-up of cluster of hatching spines showing serosa tearing from surface.

While examining the serosa covering the spiracular pits of specimens that had been poised to hatch, we noted unexpected clusters of variously sized spheroid objects on many specimens that may have a bearing on the source of the so-called hatching enzyme that is said to suddenly appear and mysteriously cause the “dissolution” of the chorion in honey bees. The spheroids occurred in the inner tracheal tubes of partly opened spiracles (Figs. 9–11 and 17), and on the surface of spiracles that had not yet opened (Figs. 18, 23–25, and 27). They could be beads of liquid coming through small openings (e.g., Fig. 13) evident in the covering of the atria in some spiracles that have not yet started to produce the liquid. If true, we suspect the liquid could be either the hatching enzyme or the source of the enzyme. The appearance of these spheroids in the SEMs is consistent with their being proteinaceous, as their appearance is comparable with that of micelles of a known protein (casein) which are likewise spherical and of comparable size (250 mm) (Spagnuolo et al. 2005). As mentioned above, we are uncertain as to whether these spheroids are covered by the serosa or are resting on the outer surface of the serosa, although the large circular object in Fig. 22 could be interpreted as one of the large spheroids having just discharged it contents exterior to the serosal surface, while Fig. 25 would serve well as a prelude to what happened in Fig. 22. In any event, the covering of the spiracular aperture in Figs. 13 and 18 consists of embryonic tissue as well as an outer layer of serosa, as evidenced by its opaque texture, similar to the partly ruptured closure in Fig. 17.

Our tentative conclusion that these spheres either may be the enzyme or may be encapsulating the enzyme that results in the dissolution of the chorion came after reviewing DuPraw’s (1967: pp. 213, 214) description of Stage 10 of the embryology of the honey bee. Although he did not know the source of the enzyme, DuPraw (p. 214) proposed that “the fragmentation of the amnion-serosa plays a role in activating the hatching enzyme,” a hypothesis supported by our observations. Hence, the small circular openings in the covering of the atrial opening in Figs. 13 and 18 and the apparent discharge of the spheres in Figs. 22–25 and 27 illustrate a plausible means by which an enzyme would be released.

Certain matters have yet to be resolved. Specimens that we examined did not reveal the fate of the outer surface of the chorion (Fig. 1). Does it undergo lysis or is it caste off earlier and our sampling missed it? This problem should be easily resolved with further observations. The appearance of the spheroids on specimens from which most of the chorion has already disappeared is difficult to understand. Why are they still there when most of the chorion is gone? However, we cannot estimate how long the presumed enzymatic material might have been emitting through the small circular holes of the covered spiracles. Furthermore, according to DuPraw (p. 213), the entire duration of Stage 10 is only 3 h perhaps making comparisons of durations of physiological events unreliable.

DuPraw (1967: p. 213) stated that the tracheal tubes of the embryo fill with air at the time the serosa fragments in the hatching process. We wonder if the gaseous filling of the tracheal tubes might be the mechanism that forces the liquid-filled tracheal tubes to flush the enzyme-laden liquid out through the atria, where it would quickly spread through the porous fibrous surface of the serosa throughout the egg. This then would account for further swelling of the body against the chorion and the leakage of the liquid over the surface of the egg as the chorion disintegrates, as pictured by Collins (2004: Fig. 1D). Simultaneous body motion would probably also assist distribution of the enzymatic fluid.

**Fig. 16.**
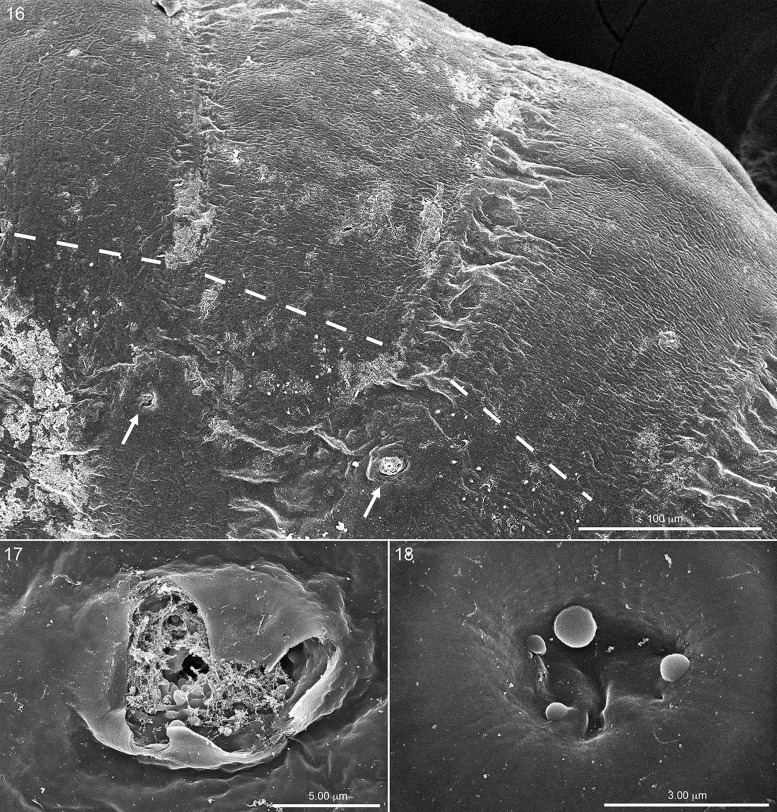
Dorsal surface of two abdominal segments of another serosa-covered first instar, lateral view, with scattered remnants of chorion. Note two spiracles identified by arrows and distribution of hatching spines below dashed line. Fig. 17. Close-up of partly opened right spiracle, on above. Fig. 18. Close-up of another serosa-cover spiracular aperture on same specimen (not visible in Fig. 16), but this spiracle not yet open though showing spheroids of possible enzyme seeming to ooze from some of the small circular openings.

One reviewer of the original manuscript of this paper was “skeptical that a few structures 1–3 µm tall (i.e., the hatching spines) are playing a mechanical role in breaking the chorion.” We agree, but if the spines primarily serve to puncture the serosa (e.g., Figs. 14 and 15) thereby allowing broad distribution of the hatching enzyme between the serosa and chorion, this might account for dissolution of the chorion. This hypothesis has some support. Hatching spines of honey bees are distributed as a broad band on each side of the first instars, which might account for the quick dissolution of the chorion. In known larvae of solitary and many cleptoparasitic bees, the spines form a linear string on each side of the first instar, so that enzymes penetrating them would dissolve a narrow line on both sides of the egg, as has been reported for numerous taxa of these bees (e.g., Rozen 1964: Fig. 5; Rozen 2016: Figs. 16 and 17).

**Fig. 19.**
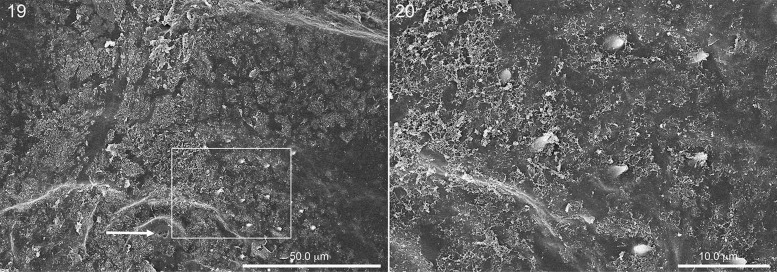
Lateral view spiracle (arrow) and surrounding area on side of abdomen of hatching first instar in which much of chorion is still present, showing spicules (hatching spines) poking through chorion. Fig. 20. Close-up of rectangle in Fig. 19.

**Fig. 21.**
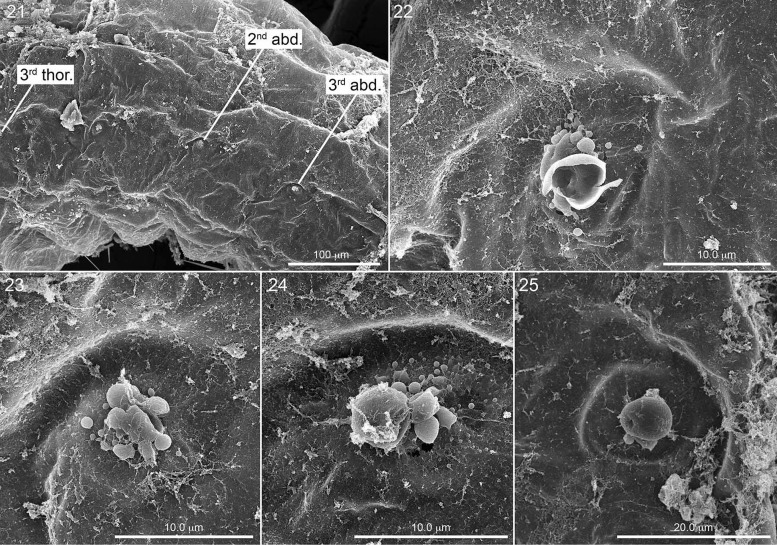
Mid-body segments showing scattered chorion debris on serosa, with three spiracles identifies by labeled arrows. Fig. 22. Third thoracic spiracle. Fig. 23. Second abdominal spiracle. Fig. 24. Third abdominal spiracle. Fig. 25. Seventh abdominal spiracle (not in Fig. 21). All close-ups exhibiting various forms of discharging spheroids.

**Fig. 26.**
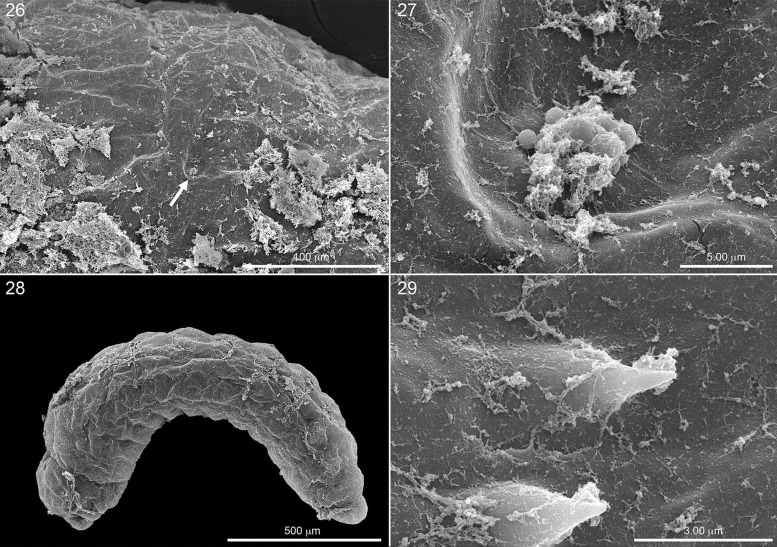
Hatching first instar, lateral view, showing discharging fourth abdominal spiracles and partial covering of fragmented chorion. Fig. 27. Close-up of spiracle. Fig. 28. Another hatching first instar, lateral view, with most of chorion gone but serosa still enveloping larva. Fig. 29. Tips of two hatching spines on abdominal segment 4 penetrating the serosa.

#### Egg Hatching of Apidae, First Instars of Which Do Not Exhibit Hatching Spines

While compiling Table 1, we noted with interest that according to Torchio (1986) the first instars of *Triepeolus dacotensis* emerged through an opening in the front of the egg as did *Epeolus compactus* according to Torchio and Burdick (1988). These observations are inconsistent with the lateral splitting of the chorion created by hatching spines. As a result, we undertook a survey of the literature to determine in what other apid taxa did hatching first instars egress through openings at the front of their chorions, as reported here in Table 2. Importantly, because many of the specimens from the original studies were deposited in the American Museum of Natural History and some were available for re-examination, we confirmed that first instars listed in boldface in Table 2 do not exhibit hatching spines. From these data we predict with some confidence that in the Nomadinae (all of which are parasitic) and parasitic Apinae there are no lateral rows of hatching spines on first instars.

**Table 2.** First instars of Apidae that on hatching egress through aperture at front of egg rather than through splitting of chorion along sides of egg taxa in boldface are those that are known to lack hatching spines because specimens originally described were re-examined for this paper

**Nomadinae**	
**Hexepeolini**	
***Hexepeolus rhodogyne* Linsley and Michener** (Rozen 1991: Figs. 7–10)	
**Epeolini**	
***Epeolus pusillus* Cresson** (Rozen and Favreau 1968)
***Epeolus ilicis* Mitchell** (Rozen 1989)
*E. compactus Cresson* (Torchio and Burdick 1988)
*T. dacotensis* (Stevens) (Torchio 1986)
***T. grandis* (Friese)** (Rozen 1991: Figs. 1–5)
**Biastini**	
*Neopasites cressoni* Crawford (Torchio et al. 1967: Fig. 9)
*Biastes emarginatus* (Schenck) (Rozen et al. 2009).
*Rhopalolemma rotundiceps* Roig-Alsina (Rozen et al. 1997: Fig. 21)
**Ammobatini:**	
*Oreopasites* (Rozen 1992).
*Parammobatodes minutus* (Mocsáry) (Rozen 2009: Figs. 18–21)
*Pseudodichroa capensis* (Friese); *P. fumipennis* Bischoff (Rozen and Michener 1968: Figs: 9–120)
**Caenoprosopidini:**	
*Caenoprosopis crabronina* Holmberg (Rozen and Roig-Alsina 1991)
**APINAE:**	
**Isepeolini:**	
**Isepeolus viperinus (Holmberg)** (Rozen 1991: Figs. 47–53)
**Osirini:**	
*Protosiris gigas* Melo** (Rozen et al. 2006)
**Protepeolini:**	
***Leiopodus singularis* (Linsley and MacSwain)** (Rozen et al. 1978: Figs. 8–14 and 22–24)
***Leiopodus lacertinus* Smith** (Roig-Alsina and Rozen 1994: Figs. 5–10).
**Tetrapedini:**	
*Coelioxoides*
**Ctenoplectrini:**	
*Ctenoplectrina*
**Rhathymini:**	
***Rhathymus bicolor* Lepeletier** (Rozen 1991: Figs. 42–46)
**Ericrocidini:**	
***Aglaomelissa duckei* (Friese)** (Rozen 1991: Figs. 54–59)
***E. lata* (Cresson)** (Rozen 1991: Figs. 64–67)
***Mesoplia rufipes* (Perty)** (Rozen 1991: Figs. 60–63)
***M. sappharina* Melo and Rocha-Filho** (Rozen et al. 2011: Figs. 24–28, 40–43, and 47)
**Melectini:**	
Melecta pacifica fulvida Cresson (Rozen 1991: Figs. 28–32)
**Melecta separata callura (Cockerell)** (Rozen 1991: Figs. 23–27)
***Thyreus lieftincki* Rozen** (Rozen 1991: Figs. 33–37)
*Xeromelecta californica* (Cresson) (Torchio and Trostle 1986, Rozen 1991: Figs. 14–22)
**Zacosmia maculata (Cresson)** (Torchio and Youssef 1968, Rozen 1991: Figs. 38–41)

Only *Protosiris gigas* Melo, identified below by**, was recognized as lacking hatching spines in the original treatment.

Regarding other families of bees, we know of no taxa where the first instar emerges from the front end of the egg. Although in Table 1 we have cited observations of lateral splitting of chorion in the Colletidae, Halictidae, Megachilidae, as well as the non-parasitic Apidae suggesting the existence of hatching spines in those families, data are insufficient to determine if there are other methods by which first instars emerge from their eggs among all groups of bees.

How does eclosion occur in the absence of lateral hatching pines? Perhaps there are as few hints: Torchio and Youssef (1968) suggested that “the first-stage larva escapes by mechanically tearing open the broader anterior tip of the egg, probably with the aid of its head spines,” i.e., the ring of cranial spines on the heads of first instar Melectini (Rozen 1991) might be involved. On noting the strongly sclerotized and spined ventral surface of the labial maxillary region on *Ericrocis lata* (Rozen 1991: Figs. 65–67) we wonder if this feature will eventually lead to an understanding of eclosion in that species.

## Conclusions

Clearly, further studies are needed to evaluate the hypotheses advanced here regarding eclosion involving hatching spines. These studies should explore the nature of the substance forming the spheroids to determine if it is proteinaceous and the enzyme, and, if so, how is it distributed so quickly to eliminate most of the chorion. We note that the fibrous surface on the serosa quickly disappears, which supports the hypothesis that its porosity functions to allow quick distribution of the hatching enzyme over the serosa to dissolve the chorion along with the fibers.

It seems likely that the spines involved in the dissolution of the chorion and serosa of the honey bee are homologous with the hatching spines of nonsocial bees both in location and apparent function. For both nonsocial bees and *A. mellifera*, the spines just above the spiracular line appear to be arranged differently where they both apparently serve to break up the chorion and destroy the serosa underneath the chorion. Studies of related social bees, such as *Bombus* and stingless honey bees (Meliponini), may yield valuable intermediates between those of known nonsocial bees and those of *A. mellifera*.
